# Ball milled phosphorus modified biochar improved *Nicotiana tabacum* L. resistance against *Phytophthora nicotianae*: reducing oxidative damage, increasing defense hormone content and promoting phenylpropanoid metabolism

**DOI:** 10.3389/fmicb.2025.1734991

**Published:** 2026-01-12

**Authors:** Chunlan Ming, Yushuai Zhang, Mengze Li, Mohamed G. Moussa, Tengfei Liu, Hang Wang, Yongfei Ma, Wuxing Huang, Zicheng Xu, Jiayang Xu, Wei Jia

**Affiliations:** 1 Key Laboratory for Tobacco Cultivation of Tobacco Industry, National Tobacco Cultivation and Physiology and Biochemistry Research Center, College of Tobacco Science, Henan Agricultural University, Zhengzhou, China; 2 Soil and Water Research Department, Nuclear Research Center, Egyptian Atomic Energy Authority, Cairo, Egypt; 3 College of Resources and Environment, Henan Agricultural University, Zhengzhou, China

**Keywords:** antioxidant enzymes, metabolites, modified biochar, phytohormones, tobacco black shank

## Abstract

**Introduction:**

Tobacco black shank (TBS), caused by *Phytophthora nicotianae*, poses a serious threat to tobacco production, highlighting the urgent need for sustainable management strategies. Meanwhile, tobacco stalk, a byproduct of tobacco cultivation, required effective recycling and value-added utilization.

**Methods:**

In this study, four types of tobacco stalk derived biochar (unmodified biochar, H_3_PO_4_-modified biochar, ball-milled biochar, and ball-milled H_3_PO_4_-modified biochar) were prepared to evaluate their efficacy in controlling TBS. We evaluated physiological indices, including chlorophyll content, antioxidant enzyme activities, reactive oxygen species levels, and phytohormone profiles, along with changes in tobacco leaf metabolites, to explore the impact of modified biochar on diseased tobacco plants.

**Results:**

The ball milled-phosphorus modified biochar (BPT) exhibited a dense and uniform pore structure, markedly increased surface P content, and introduced abundant -OH and P-O functional groups, resulting in the most effective disease suppression. Soil application of BPT significantly reduced lesion length in infected plants, enhanced chlorophyll content, increased the activities of antioxidant enzymes [catalase (CAT), peroxidase (POD), and superoxide dismutase (SOD)], decreased malondialdehyde (MDA), hydrogen peroxide (H_2_O_2_), and superoxide anion (O_2_^−^) levels. Moreover, BPT modulated phytohormone levels elevating indole-3-acetic acid (IAA), jasmonic acid (JA), abscisic acid (ABA), and salicylic acid (SA) and reshaped amino acid, lipid, and phenolic acid profiles. Metabolic pathway analysis indicated that BPT promoted phenylpropanoid as well as phenylalanine, tyrosine, and tryptophan biosynthesis, thereby strengthening tobacco resistance to *P. nicotianae*.

**Discussion:**

This study elucidate the mechanisms underlying biochar-induced disease resistance and provide a promising approach for sustainable management of tobacco black shank using modified biochar.

## 1 Introduction

Soil borne pathogens are among the most destructive plant pathogens due to their transmission through the soil. These diseases severely damage root systems and can cause unpredictable effects on the aerial parts of plants, including stem rot, tissue discoloration, leaf wilting, and even plant death (Bhatt et al., 2024). Tobacco black shank (TBS), caused by *Phytophthora nicotianae* (*P. nicotianae*) is a representative example of such threats. *Phytophthora nicotianae* can survive in soil for long periods and infect tobacco plants at nearly any growth stage, resulting in root rot, blackened stems, wilted, and significant yield losses (Wang et al., 2022; Han et al., 2016). Current TBS management strategies include crop rotation, the use of resistant varieties, chemical fungicides, and biological controls. Although chemical fungicides remain the most widely used approach, their excessive application leads to environmental contamination, chemical residues, and the development of fungicide-resistant strains (Wang et al., 2020). The cultivation of resistant varieties and biological control methods, while effective, are costly and time-consuming (Ma et al., 2024; Yu et al., 2024). Therefore, developing green, safe, and cost-effective strategies for TBS control is urgently needed.

China is the world’s largest producer of tobacco, ranking first in both cultivated area and yield. During tobacco production and processing, large quantities of tobacco residues such as stalk are generated (Liu et al., 2015). These residues are often burned or dumped, causing environmental pollution and resource waste (Banožić et al., 2020). Consequently, finding sustainable approaches to utilize tobacco stalks has become an important environmental and economic concern.

In recent years, biochar (BC) derived from pyrolyzed tobacco stalks has attracted growing attention (Yu et al., 2021; Yang et al., 2022; Zhao et al., 2024). BC is a carbon-rich, porous material produced from the pyrolysis of organic biomass (e.g., crop residues, manure) under limited oxygen conditions (Azeem et al., 2021). As a soil amendment, BC improves soil structure, enhances nutrient and water retention and reduces nutrient leaching. Studies have demonstrated that BC can increase crop yields, and its beneficial effects tend to accumulate over time (Major et al., 2010). For example, BC improved quinoa yield and quality by enhancing soil moisture and nutrients (Daraei et al., 2024), and it promoted the abundance and activity of soil microbial communities, which directly support plant growth (Ding et al., 2016; Ng et al., 2022). BC has also shown effectiveness in mitigating abiotic stresses such as heavy metal toxicity and drought. BC immobilized Cu, Pb, Cd, and Zn in soil, thereby reducing their uptake by plants and increasing biomass (Qian et al., 2024). Similarly, Kumar et al. (2024) found that BC application enhanced the shoot and root biomass of coriander and Bengal gram under drought stress by improved soil structure and moisture retention.

However, despite its carbon-rich composition, BC often contains limited nutrients and functional groups. Thus, BC modification through chemical, physical or biological means can enrich its surface properties, enhance stability and improve performance (Hafeez et al., 2022). Physical ball milling, as a green and efficient method for biochar modification, enables unprocessed biochar to develop a more uniform pore structure and a greater abundance of surface functional groups, thereby enhancing its thermal stability and cation exchange capacity. For instance, Quan et al. (2025) evealed that ball milling led to the formation of additional -OH and -COOH functional groups on the surface of corn straw biochar, which subsequently enhanced its adsorption capacity for ammonium nitrogen. Zhang et al. (2025) indicated that ball milling increased the specific surface area and surface functional groups of straw biochar and enhanced adsorption performance. Chemically modified biochar shows potential in plant growth and plant disease suppression. Phosphorus-modified BC has been shown to be effective in enhancing soil phosphorus levels, thus facilitating plant growth (Mbasabire et al., 2024). phosphorus-modified BC also significantly increased soil nutrients content, while reducing Cd and Pb translocation to lettuce (Han et al., 2023). BC containing phosphate has also been used as a slow-release fertilizer to enhance soil nutrients and fertility (Ding et al., 2016). In the case of co-modified biochar, ball milled phosphorus-loaded BC effectively adsorbed heavy metals, improving soil conditions for plant growth (Zhang et al., 2021). However, the effect of co-modified biochar on plant disease resistance has received limited attention.

To achieve the dual goals of sustainable utilization of tobacco stalks and ecological management of tobacco black shank (TBS), this study focused on developing and evaluating modified biochar derived from tobacco stalks. One pristine biochar, three types of modified biochar H_3_PO_4_-modified, CaCl_2_-modified, and MgCl_2_-modified tobacco stalk biochar’s along with their corresponding ball-milled variants, were prepared and assessed for their efficacy in suppressing TBS caused by *P. nicotianae*. Among these, the ball-milled H_3_PO_4_-modified biochar exhibited the most effective disease control performance. Based on this finding, four representative biochars unmodified tobacco stalk biochar, H_3_PO_4_-modified biochar, ball-milled modified biochar, and ball-milled H_3_PO_4_-modified biochar were selected for further investigation. The study aimed to 1) Evaluate the influence of different biochar types on TBS incidence and tobacco plant health; 2) Elucidate the physiological and metabolic mechanisms by which biochar enhances tobacco resistance to *P. nicotianae*; and 3) Provide an eco-friendly and value-added approach for recycling tobacco waste into a sustainable disease management material. The outcomes of this research not only promote the effective reuse of tobacco processing byproducts but also contribute to the development of a green, safe, and cost-effective strategy for controlling TBS in tobacco cultivation systems.

## 2 Materials and methods

### 2.1 Preparation for biochar

Tobacco stalks were collected from the Nanyang tobacco-growing region, Henan Province, China. The stalks were washed thoroughly with deionized water, air-dried, and ground into fine particles. Four types of biochar and their derivatives were prepared as follows: 1) Tobacco stalk biochar (T): Crushed stalks were pyrolyzed in a tube furnace (Model TL1200, BEQ, Anhui, China) at 600 °C for 120 min under a nitrogen flow of 0.2 L /min, with a heating rate of 10 °C/min. The resulting biochar yield was approximately 25.3%; 2) Ball-milled biochar (BT): A 5 g portion of T was milled using a planetary ball mill (Model XQM-0.4A, Tencan Power, Changsha, China) with stainless steel grinding jars (500 mL) and balls (diameter: 5 mm, ball-to-powder ratio 20:1). The milling process was conducted at 500 rpm for 120 min; 3) Phosphoric acid-modified biochar (PT): Five grams of T were mixed with 40 mL of 85% H_3_PO_4_ in a hydrothermal reactor and activated at 200 °C for 720 min; and 4) Ball-milled phosphoric acid-modified biochar (BPT): The prepared PT was further processed by ball milling under the same conditions used for BT. Additionally, the preparation procedures for CaCl_2_- and MgCl_2_-modified biochars, and their ball-milled variants, are described in Supplementary Text S1.

### 2.2 Tobacco pot experiment

Seeds of flue-cured tobacco (*Nicotiana tabacum* L., cultivar K326) were provided by Henan Agricultural University. The physicochemical properties of the experimental soil are listed in Supplementary Text S2. The *P. nicotianae* isolate (XC-26-5) was provided from College of Plant Protection, Henan Agricultural University and has been deposited at the China Center for Type Culture Collection (CCTCC) under the accession number CCTCC M 2024037. The strain was cultured on oatmeal agar (OMA) at 28 °C in the dark. Tobacco seeds were surface-sterilized in 10% (v/v) NaClO for 3–5 min, followed by 75% ethanol for 30 s, rinsed three to four times with sterile water, and soaked for 8 h. The sterilized seeds were germinated in seedling trays filled with sterilized substrate soil. At the four-leaf-one-heart stage, uniform seedlings were transplanted into pots containing 2 kg of soil mixed with biochar (10 g) at a rate of 0.5% (w/w) following Jaiswal et al. (2014). Each pot contained one plant.

Eight types of biochar were initially tested include T (unmodified), CaT (CaCl_2_-modified), MgT (MgCl_2_-modified), PT (H_3_PO_4_-modified), BT (ball-milled), BCaT (ball-milled CaCl_2_-modified), BMgT (ball-milled MgCl_2_-modified), and BPT (ball-milled H_3_PO_4_-modified). After 30 days of transplantation, plant growth was observed (Supplementary Figure S2), and using a sterilized puncher, *P. nicotianae* mycelial plugs (5 mm in diameter) were inoculated onto the stem surface (Ma et al., 2024). Seven days of post-inoculation, the stem bases were observed for blackening to confirm successful infection by *P. nicotianae*, and lesion lengths were measured, and the middle leaves were collected and stored at −80 °C for further analyses. Based on the superior growth and disease suppression observed in PT and BPT treatments (Supplementary Figure S3), these two, along with T and BT, were selected for subsequent physiological and biochemical investigations. The experiment comprised 10 treatments divided into two groups: 1) Non-inoculated: N-CK, N-T, N-BT, N-PT, N-BPT and 2) Inoculated: CK, T, BT, PT, BPT. Each treatment included three biological replicates arranged in a completely randomized design.

### 2.3 Physico-chemical characterization analysis biochar

The surface morphology of T, BT, PT, and BPT were characterized using scanning electron microscopy (SEM). Functional groups were identified by Fourier transform infrared spectroscopy (FTIR), surface elemental composition was analyzed by X-ray photoelectron spectroscopy (XPS) and Brunauer–Emmett–Teller (BET) was used for the detection of specific surface area and pore structure of biochars. Detailed instrument specifications are provided in Supplementary Text S3.

### 2.4 Determination of chlorophyll content

Fresh leaf samples (0.5 g) were immersed in 25 mL 95% ethanol and incubated in darkness for 24–36 h until the leaves became completely bleached. The absorbance of the extract was measured at 665 nm and 649 nm using an enzyme-linked immunosorbent assay reader, and chlorophyll a, b, and total chlorophyll contents were calculated as described by Sun et al. (2022). Each treatment was repeated three times.

### 2.5 Determination of antioxidant system and hormone content

Leaves from each treatment were collected to determine the activities of catalase (CAT), peroxidase (POD), and superoxide dismutase (SOD), as well as the levels of reactive oxygen species (ROS) indicators, including malondialdehyde (MDA), superoxide anion (O_2_^.-^), hydrogen peroxide (H_2_O_2_). All parameters were measured using commercial assay kits (Suzhou Comin Biotechnology Co., Ltd., China). Phytohormone levels including abscisic acid (ABA), indole-3-acetic acid (IAA), jasmonic acid (JA), and salicylic acid (SA) were quantified using enzyme-linked immunosorbent assay (ELISA) kits according to the manufacturer’s instructions. Each treatment was repeated three times. The detailed parameters of the kits are provided in Text S4.

### 2.6 Metabolomics analysis

Approximately 100 mg of tobacco leaf tissue was mixed with 500 μL of 80% methanol in an Eppendorf tube. The samples were vortexed, kept on ice for 5 min, and centrifuged at 15,000 × g for 20 min at 4 °C. The supernatant was diluted, re-centrifuged, and the final extract was subjected to liquid chromatography-mass spectrometry (LC–MS) analysis for metabolite profiling. Six replicates were performed for each treatment. Quality control (QC) samples were prepared by taking equal volumes from each test sample and mixing them uniformly, and repeating three times. The specific instrument model and detailed parameters for LC–MS are provided in Supplementary Text S5. The raw data files generated by UHPLC–MS/MS were processed using the Compound Discoverer 3.3 (CD3.3, ThermoFisher) to perform peak alignment, peak picking, and quantitation for each metabolite. These metabolites were annotated using a local metabolic database. Principal component analysis (PCA) and partial least squares discriminant analysis (PLS-DA) were performed on the metabolic data. The metabolites with VIP > 1 and *p* < 0.05 and FC > 1.5 or < 0.67 were considered to be differential metabolites.

### 2.7 Statistical analysis

FTIR spectra and XPS data were processed using Origin is a graphing software. Statistical analyses were conducted using SPSS 26.0, and results are expressed as mean ± standard error (SE) of three replicates. Statistical differences between the treatment means were determined by one-way ANOVA followed by the Tukey’s HSD *post-hoc* test at a significance level of 0.05. Data visualization was performed using GraphPad Prism 9.5.

## 3 Results

### 3.1 Biochar characterization

#### 3.1.1 SEM analysis and elemental compositions

**Figure 1:**
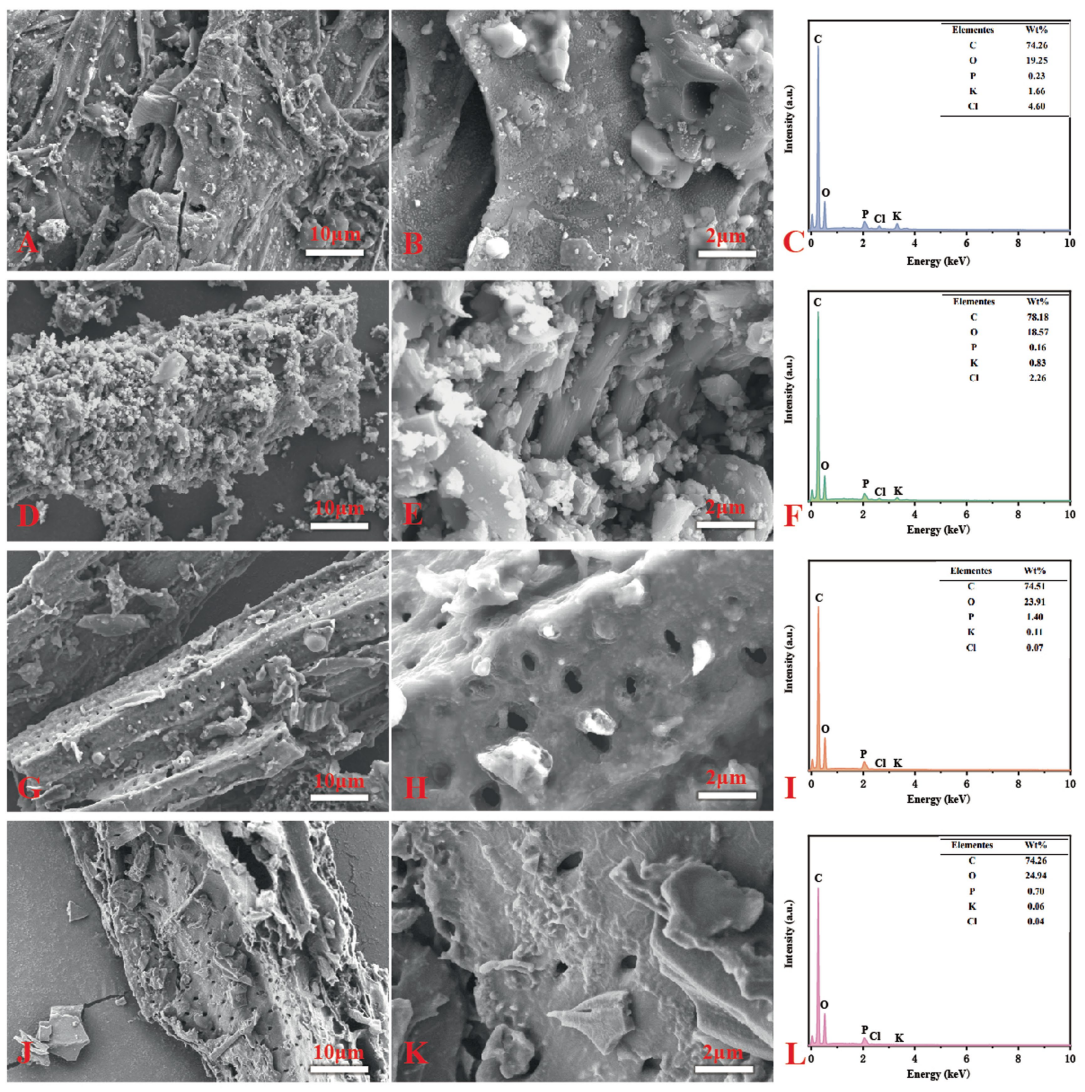
SEM images of T (unmodified biochar), BT (ball-milled biochar), PT (H_3_PO_4_-modified biochar), and BPT (ball-milled H_3_PO_4_-modified biochar) at different magnifications and surface element concent analysis (**A-C**: T; **D-F**: BT; **G-I**: PT; **J-L**: BPT).

Scanning electron microscopy (SEM) revealed pronounced differences in surface morphology and elemental composition among the various biochar types (Figure 1). The pristine tobacco stalk biochar (T) displayed a relatively smooth surface with limited pore development (Figures 1A,B). In contrast, ball milling (BT) generated abundant surface particles and markedly enhanced porosity (Figures 1D,E). Phosphoric acid modification (PT) and the combined treatment of phosphoric acid with ball milling (BPT) further increased surface roughness and produced more uniform and well-developed pore structures (Figures 1G,H,J,K). Elemental mapping confirmed that H_3_PO_4_ modification substantially enriched the biochar surface with oxygen (O) and phosphorus (P), the P on the surface of the biochar increased from T (0.23%) to PT (1.40%), and from BT (0.16%) to BPT (0.70%) (Figures 1C,F,I,L).

#### 3.1.2 BET analysis of biochar

The pore size distribution and N_2_ adsorption–desorption isotherms of T. BT, PT and BPT were measured by BET (Supplementary Figure S1). According to the IUPAC classification, the N_2_ adsorption–desorption isotherms of the four biochars correspond to Type III, while the hysteresis loops observed for T and BT at relative pressure range of 0.4–0.9 exhibit an H3 type (Nguyen et al., 2023). The pore-size distribution curves indicate that four biochars were characterized by mesoporous structures (2–50 nm), with the peak of the pore size distribution for BPT located at approximately 4 nm. In Supplementary Table S1, ball milling modification increased the specific surface area of the raw biochar and reduced the average pore size, whereas phosphoric acid modification increased the average pore size while decreasing the specific surface area. This phenomenon, combined with the increased phosphorus loading in the phosphoric acid-modified biochar, suggests that phosphate groups may have blocked the pores of the biochar (Sathasivam et al., 2025).

#### 3.1.3 FTIR spectrum of biochar

**Figure 2:**
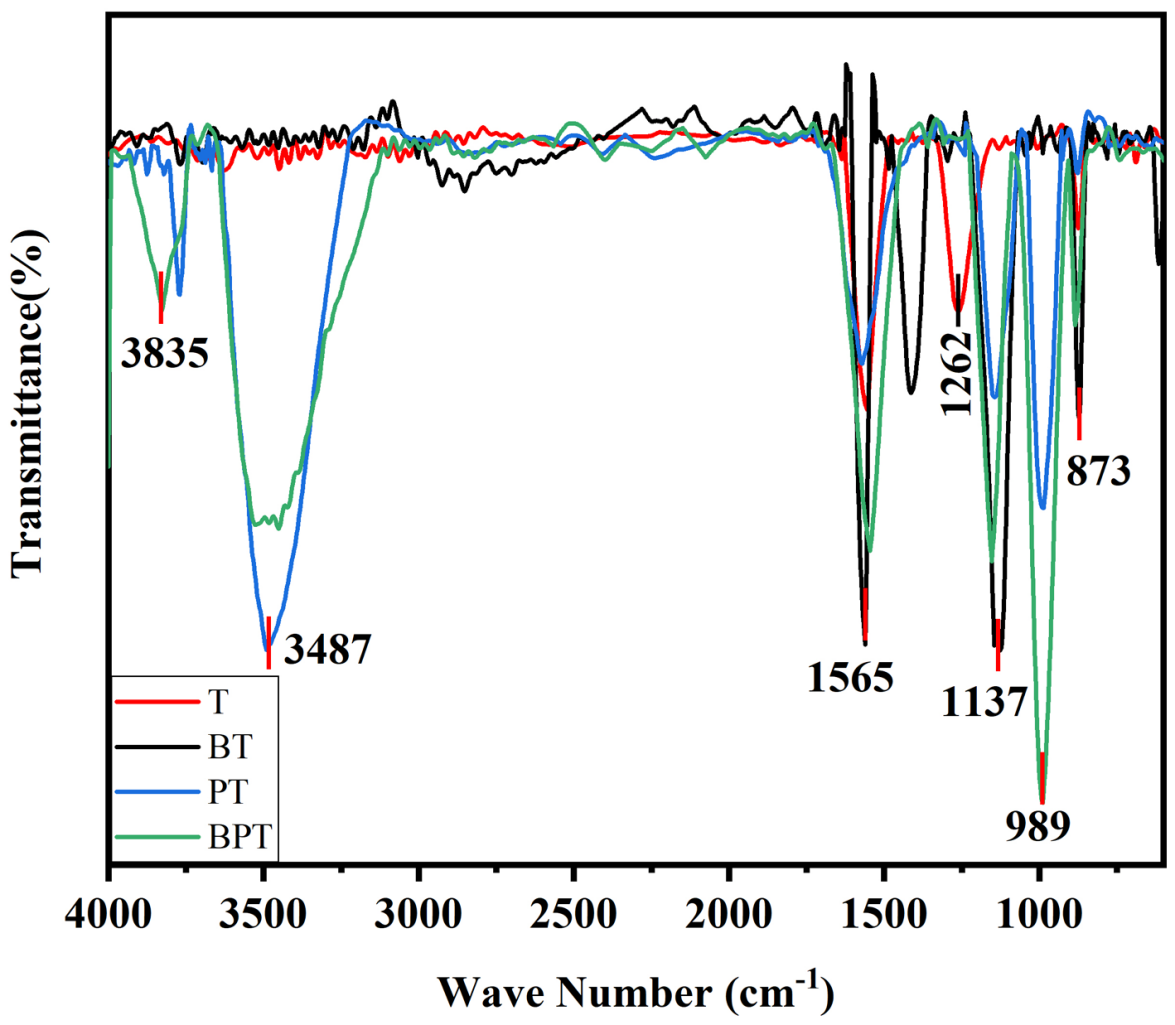
The FTIR spectral analysis of different biochar’s. T (unmodified biochar), BT (ball-milled biochar), PT (H_3_PO_4_-modified biochar), and BPT (ball-milled H_3_PO_4_-modified biochar).

The FTIR spectra revealed distinct in surface functional groups among the four biochar samples, while several characteristic peaks were commonly observed at approximately 1,565, 1,137, and 873 cm^−1^ (Figure 2). The absorption band at 1565 cm^−1^ corresponds to the C=C stretching vibration of aromatic compounds (Zhang et al., 2023). Peaks within the range of 1,262–1,137 cm^−1^ are associated with the vibrations of P-O-P, P-O-C, and P-OH bonds (Yang et al., 2025). The band near 873 cm^−1^ is attributed to C-H bending vibration. In addition, prominent peaks round 3,487 cm^−1^ and 989 cm^−1^, observed in both PT and BPT samples, correspond to the stretching vibrations of -OH and P-O groups, respectively (Du et al., 2025; Rizwan et al., 2020). These spectral features confirm the successful incorporation of -OH and phosphate functional group onto the biochar surface through phosphoric acid modification.

#### 3.1.4 XPS analysis of different biochar

**Figure 3:**
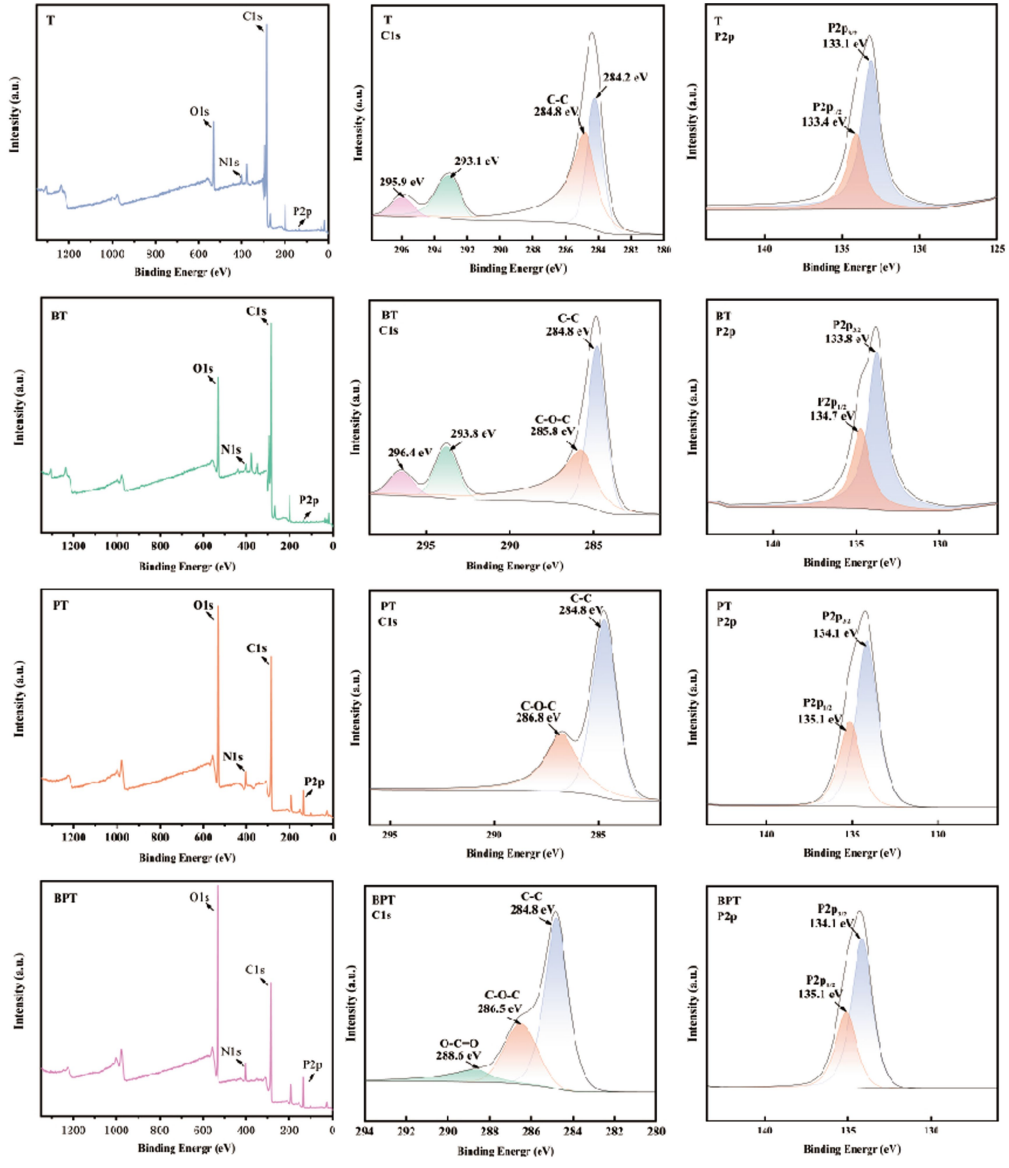
The XPS analysis of different biochar. T (unmodified biochar), BT (ball-milled biochar), PT (H_3_PO_4_-modified biochar), and BPT (ball-milled H_3_PO_4_-modified biochar).

X-ray photoelectron spectroscopy (XPS) was employed to characterize the surface elemental composition and chemical states of the four biochar. Strong C and O peaks were detected in all samples, whereas weak P peaks appeared in the H_3_PO_4_-modified biochar (PT and BPT), confirming successful phosphorus incorporation (Figure 3). The C1s and P2p peaks spectra of each biochar was deconvoluted by peak fitting to identify specific bonding environments. For the unmodified samples (T and BT), the C1s spectra were resolved into four components, primarily corresponding to C-C (284.8 eV) and C-O-C (285.8 eV) bonds. After H_3_PO_4_ modification, the C1s spectrum of PT displayed two major peaks at 284.8 eV and 286.8 eV, attributed to C-C and C-O-C bonds, respectively. In the BPT sample, an additional peak appeared at 288.6 eV, assigned to O-C=O groups, indicating partial surface oxidation induced by phosphorus modification and ball milling. The P2p spectra of PT and BPT exhibited two characteristic peaks between 133.1 and 135.1 eV, corresponding to P-O and C-P-O bonds, respectively. These results further confirm the formation of phosphate-related functional groups on the biochar surface following H_3_PO_4_ treatment and the enhanced incorporation efficiency achieved through ball milling.

### 3.2 Control effect of biochar on tobacco black shank

**Figure 4:**
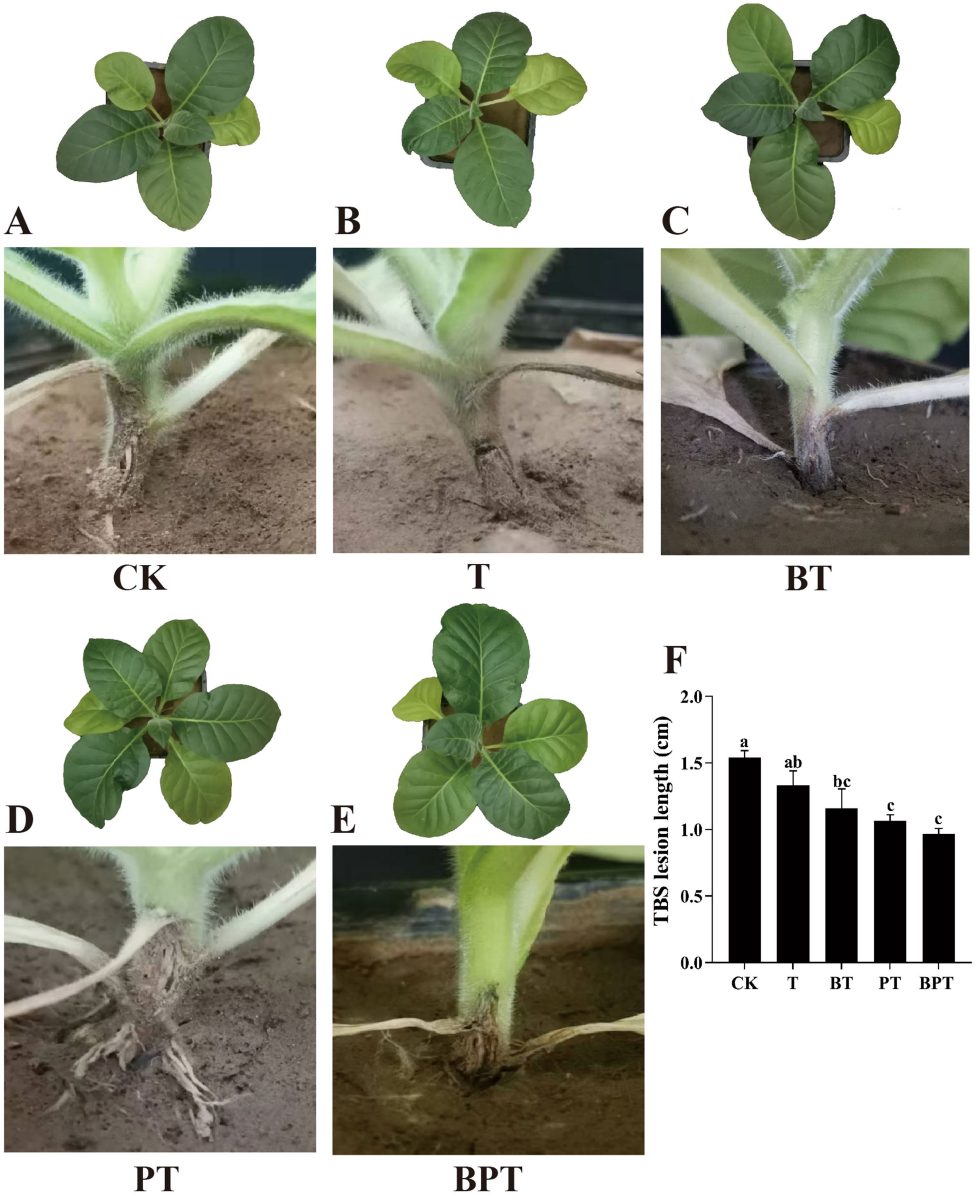
The phenotype **(A-E)** and lesion length **(F)** of tobacco plants under different treatments. CK is the control (**A**, no biochar), and T (**B**, unmodified biochar), BT (**C**, ball-milled biochar), PT (**D**, H_3_PO_4_-modified biochar), BPT (**E**, ball-milled H_3_PO_4_-modified biochar) are the treatments. Data are presented as mean ± SE (*n* = 3). Different letters indicate significant differences among treatments at *P* < 0.05.

Our results in (Figure 4F) illustrates the effect of different biochar treatments on lesion length caused by tobacco black shank (TBS). All biochar treatments significantly reduced lesion length compared with the control. Among them, the BPT and PT treatments achieved the greatest reductions, decreasing lesion length by 37.01 and 31.17%, respectively. Significant differences were observed among the biochar types, with the BPT treatment exhibiting 8.49, 16.38, and 27.07% shorter lesion lengths than PT, BT, and T, respectively.

### 3.3 Biochar increases chlorophyll content in tobacco

**Figure 5:**
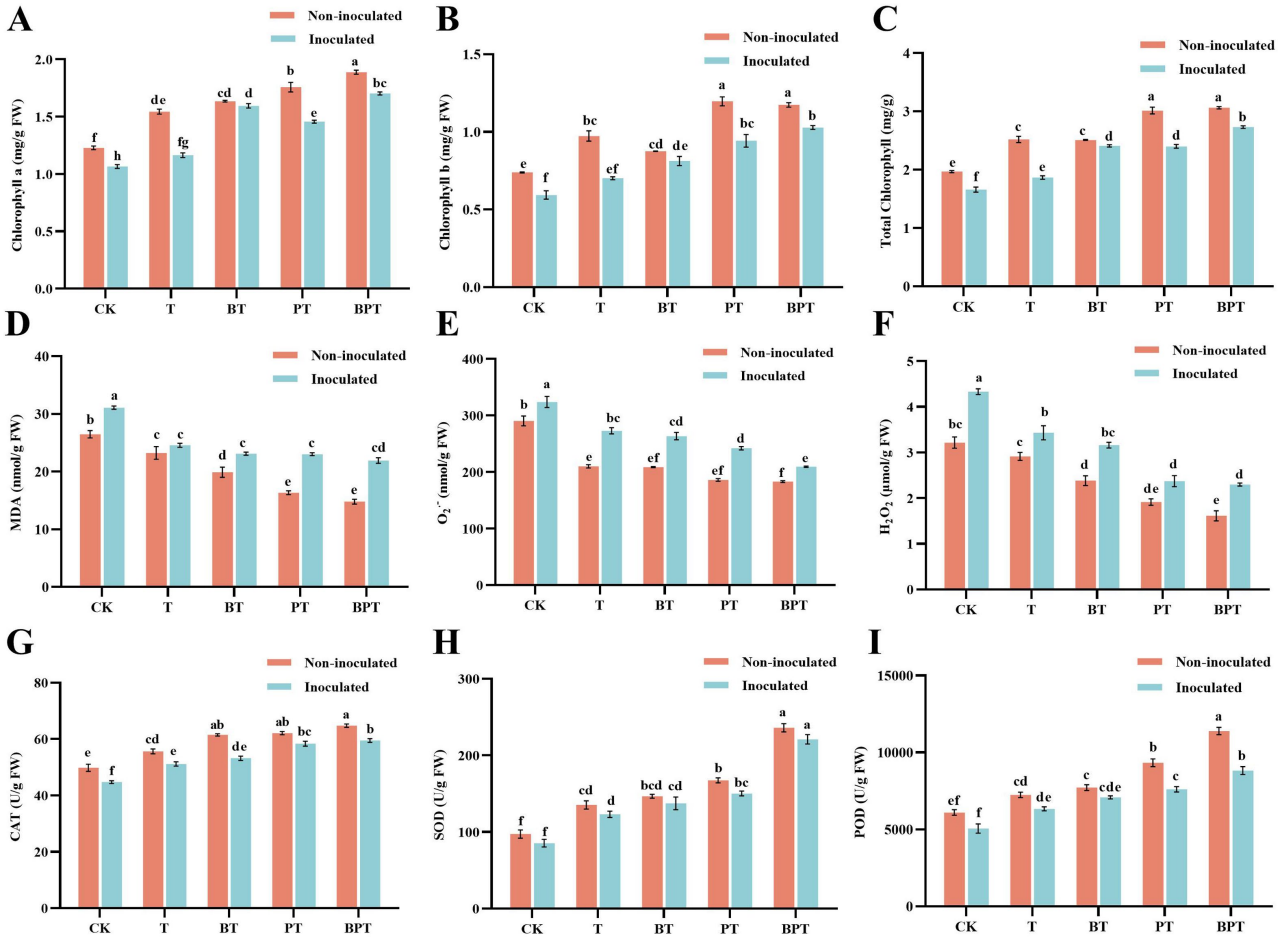
Effects of different treatments on physiological and biochemical parameters of tobacco. **(A)** Chlorophyll *a* content; **(B)** Chlorophyll *b* content; **(C)** Total chlorophyll content; **(D)** Malondialdehyde (MDA) content; **(E)** Superoxide anion (O_2_.-) content; **(F)** Hydrogen peroxide (H_2_O_2_) content; **(G)** Catalase (CAT) activity; **(H)** Superoxide dismutase (SOD) activity; **(I)** Peroxidase (POD) activity. Data are presented as mean ± SE (*n* = 3). Different letters indicate significant differences among treatments at *P* < 0.05.

The effects of different biochar treatments on chlorophyll accumulation in tobacco are presented in Figures 5A–C. In the non-inoculated group, the application of ball-milled phosphorus-modified biochar (N-BPT) significantly increased chlorophyll *a*, chlorophyll *b*, and total chlorophyll contents by 53.70, 58.90, and 55.65%, respectively, compared with the non-inoculated control (N-CK). Under *P. nicotianae* inoculation, chlorophyll contents in plants treated with BPT were markedly higher than those in CK, T, BT, and PT treatments. Specifically, chlorophyll *a* content in BPT increased by 59.74, 46.22, 6.78, and 16.89%, while chlorophyll *b* content increased by 73.23, 46.58, 26.55, and 9.06%, respectively. However, compared with the non-inoculated BPT treatment, total chlorophyll, chlorophyll *a*, and chlorophyll *b* levels in the inoculated BPT group decreased by 10.85, 14.17, and 12.10%, respectively. These findings indicate that *P. nicotianae* infection reduced chlorophyll content in tobacco leaves, but the application of BPT effectively mitigated this decline and maintained higher chlorophyll levels under pathogen stress.

### 3.4 Effect of biochar on the antioxidant system of tobacco

Figures 5D–F present the changes in reactive oxygen species (ROS) levels in tobacco under different treatments. Inoculation with *P. nicotianae* significantly increased the accumulation of malondialdehyde (MDA), superoxide anion (O_2_^·-^), and hydrogen peroxide (H_2_O_2_) compared with the non-inoculated group, indicating enhanced oxidative stress. However, all biochar treatments markedly reduced the levels of these ROS-related indicators. In the inoculated plants, compared with the infected control (CK), the T, BT, PT, and BPT treatments decreased MDA content by 26.52, 34.52, 35.02, and 41.80%, respectively; O_2_^·-^ content by 18.71, 22.91, 33.84, and 54.76%; and H_2_O_2_ content by 20.80, 27.04, 45.20, and 46.97%. These results demonstrate that *P. nicotianae* infection triggered oxidative damage in tobacco seedlings, whereas biochar application particularly BPT effectively mitigated ROS accumulation. Additionally, Figures 5G-I show the activities of the antioxidant enzymes CAT, SOD, and POD across treatments. Overall, enzyme activities were reduced in inoculated plants compared with the non-inoculated controls. Within each group, enzyme activity followed the order: BPT > PT > BT > T > CK. Relative to N-CK, the activities of CAT, SOD, and POD in CK decreased by 11.32, 20.47, and 13.87%, respectively. In contrast, compared with CK, the T, BT, PT, and BPT treatments increased POD activity by 25.22, 39.87, 50.30, and 74.31%; SOD activity by 44.08, 60.88, 75.86, and 158.71%; and CAT activity by 14.37, 18.88, 30.41, and 32.93%, respectively. Collectively, these results indicate that biochar application, particularly the ball-milled phosphorus-modified biochar (BPT), effectively reduced oxidative stress by lowering ROS accumulation and enhancing the activities of key antioxidant enzymes in tobacco under *P. nicotianae* infection.

### 3.5 Effect of biochar on phytohormone contents in tobacco plants

**Figure 6:**
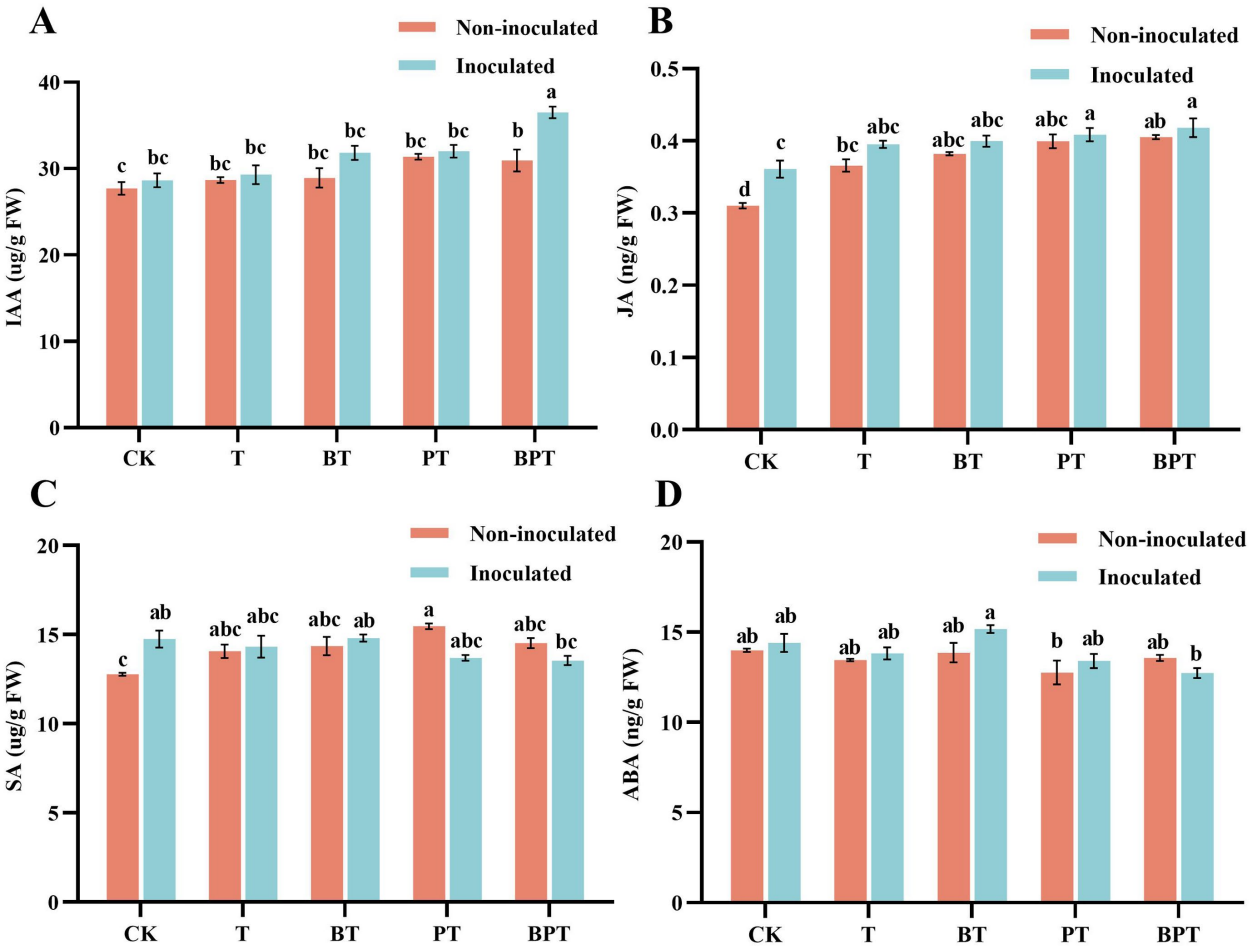
Effects of different biochar treatments on hormone contents in tobacco. **(A)** Indole-3-acetic acid (IAA) content; **(B)** Jasmonic acid (JA) content; **(C)** Salicylic acid (SA) content; **(D)** Abscisic acid (ABA) content. Data are presented as mean ± SE (*n* = 3). Different letters indicate significant differences among treatments at *P* < 0.05.

Biochar amendment markedly influenced the phytohormone profiles of tobacco seedlings (Figure 6). In general, the levels of indole-3-acetic acid (IAA), jasmonic acid (JA), and abscisic acid (ABA) were higher in the inoculated group than in the non-inoculated group. Among non-inoculated treatments, JA content was lowest in N-CK (0.31 ng/g) and highest in N-BPT (0.40 ng/g), representing a 30.56% increase compared with N-CK. Following inoculation, JA content in CK increased by 16.29% relative to N-CK, while the BPT treatment exhibited the highest JA level, which was 34.79, 15.91, and 3.24% higher than those in N-CK, CK, and N-BPT, respectively. No significant difference in JA content was observed between PT and BPT after inoculation. The variation pattern of IAA was like that of JA, following the order BPT > PT > BT > T > CK. In contrast, the salicylic acid (SA) and ABA contents in the BPT treatment were lower than those in the other treatments. These findings suggest that biochar amendments, particularly the ball-milled phosphorus-modified biochar (BPT), modulated phytohormone homeostasis in tobacco, promoting the accumulation of defense-related hormones (JA and IAA) while fine-tuning SA and ABA levels to enhance resistance against *P. nicotianae* infection.

### 3.6 Biochar alters the metabolic profile of tobacco plants

**Figure 7:**
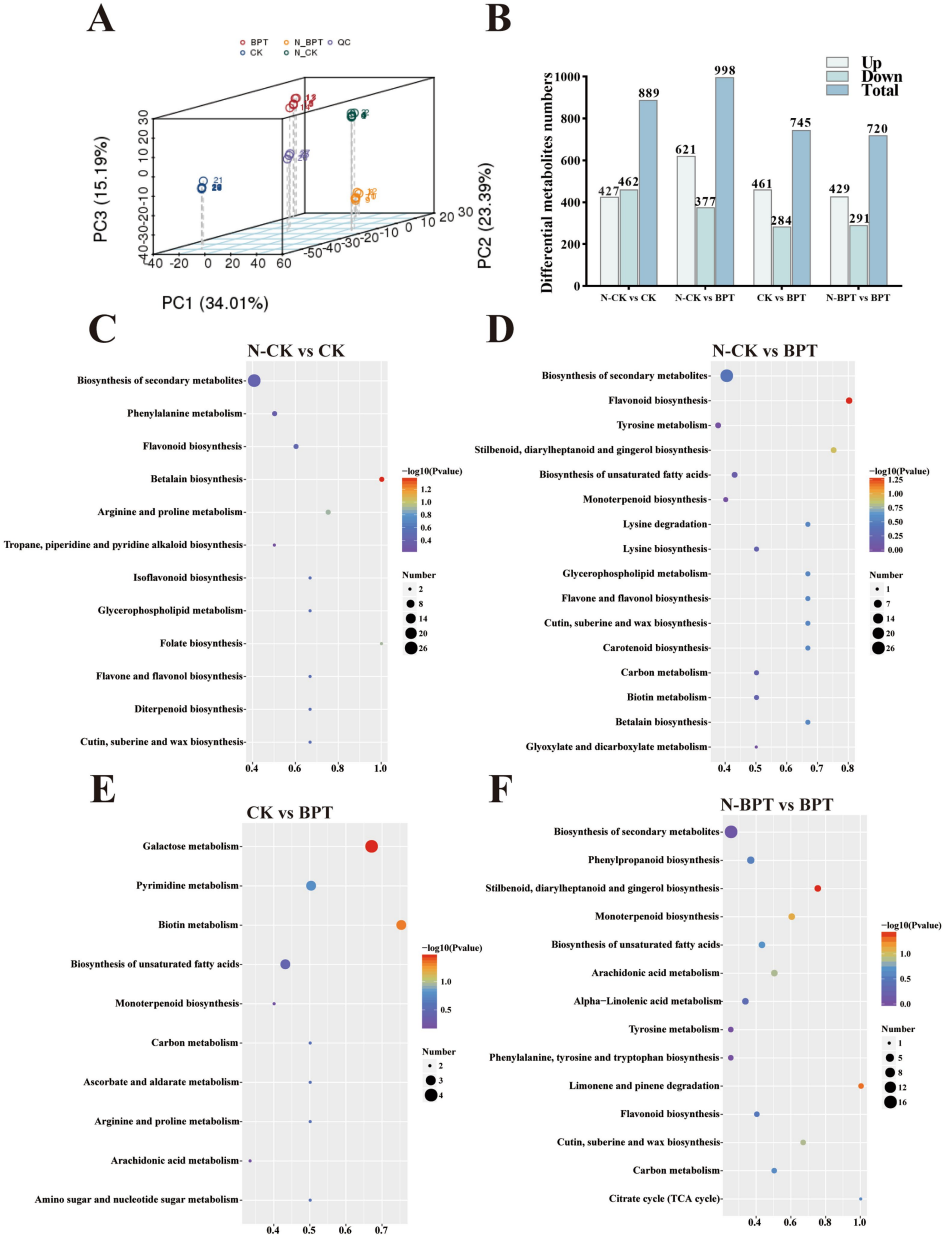
Effects of different treatments on tobacco metabolism. N-CK is the control (no biochar, non inoculated), and CK (no biochar, inoculated), N-BPT (ball-milled H_3_PO_4_-modified biochar, non inoculated), BPT (ball-milled H_3_PO_4_-modified biochar, inoculated) are the treatments. **(A)** PCA analysis. **(B)** Statistical analysis of differential metabolites. **(C-F)** KEGG enrichment bubble diagram.

To elucidate the metabolic mechanisms underlying the effects of biochar on TBS-infected tobacco plants, untargeted metabolomic analyses were performed using BPT as the representative biochar treatment. Both non-inoculated (N-CK and N-BPT) and inoculated (CK and BPT) groups were analyzed. Supplementary Figure S4 shows the classification of metabolites identified in tobacco leaves. Among them, lipids and lipid-like molecules constituted the largest category (35.84%), followed by organic acids and derivatives (15.53%), organoheterocyclic compounds (12.88%), organic oxygen compounds (11.62%), and phenylpropanoids and polyketides (10.63%). Principal component analysis (PCA) revealed clear separation among the four treatment groups, explaining 34.01, 23.39, and 15.19% of the total variation by the first three principal components, respectively (Figure 7A). The close clustering of biological replicates within each group indicated high data reproducibility, while distinct separation among treatments demonstrated significant metabolic differentiation. According to PLS-DA (Supplementary Figure S5), all comparison groups met the criteria of R2 > Q2, and the regression line intercept of Q2 was less than 0, indicating that the PLS-DA model was stable, reliable, and free from overfitting. Figure 7B revealed 889 differential metabolites were detected between N-CK and CK (427 upregulated, 462 downregulated), 998 between N-CK and BPT (621 upregulated, 377 downregulated), 745 between CK and BPT (461 upregulated, 284 downregulated), and 720 between N-BPT and BPT (429 upregulated, 291 downregulated). The N-CK vs. BPT comparison exhibited the highest number of differential metabolites, whereas the N-BPT vs. BPT comparison showed the fewest, indicating greater metabolic similarity between N-BPT and BPT treatments. KEGG pathway enrichment analysis of differential metabolites revealed distinct metabolic responses among treatments (Figures 7C–F). In the N-CK vs. CK comparison, differential metabolites were mainly enriched in betalain biosynthesis, flavonoid biosynthesis, arginine and proline metabolism, and secondary metabolite biosynthesis. In contrast, the N-CK vs. BPT comparison showed enrichment in flavonoid biosynthesis, stilbenoid, diarylheptanoid, and gingerol biosynthesis, tyrosine metabolism, and secondary metabolite biosynthesis. The CK vs. BPT group exhibited significant enrichment in galactose metabolism, biotin metabolism, and pyrimidine metabolism. For the N-BPT vs. BPT comparison, enriched pathways included stilbenoid, diarylheptanoid and gingerol biosynthesis, monoterpenoid biosynthesis, phenylpropanoid biosynthesis, and tyrosine metabolism.

**Figure 8:**
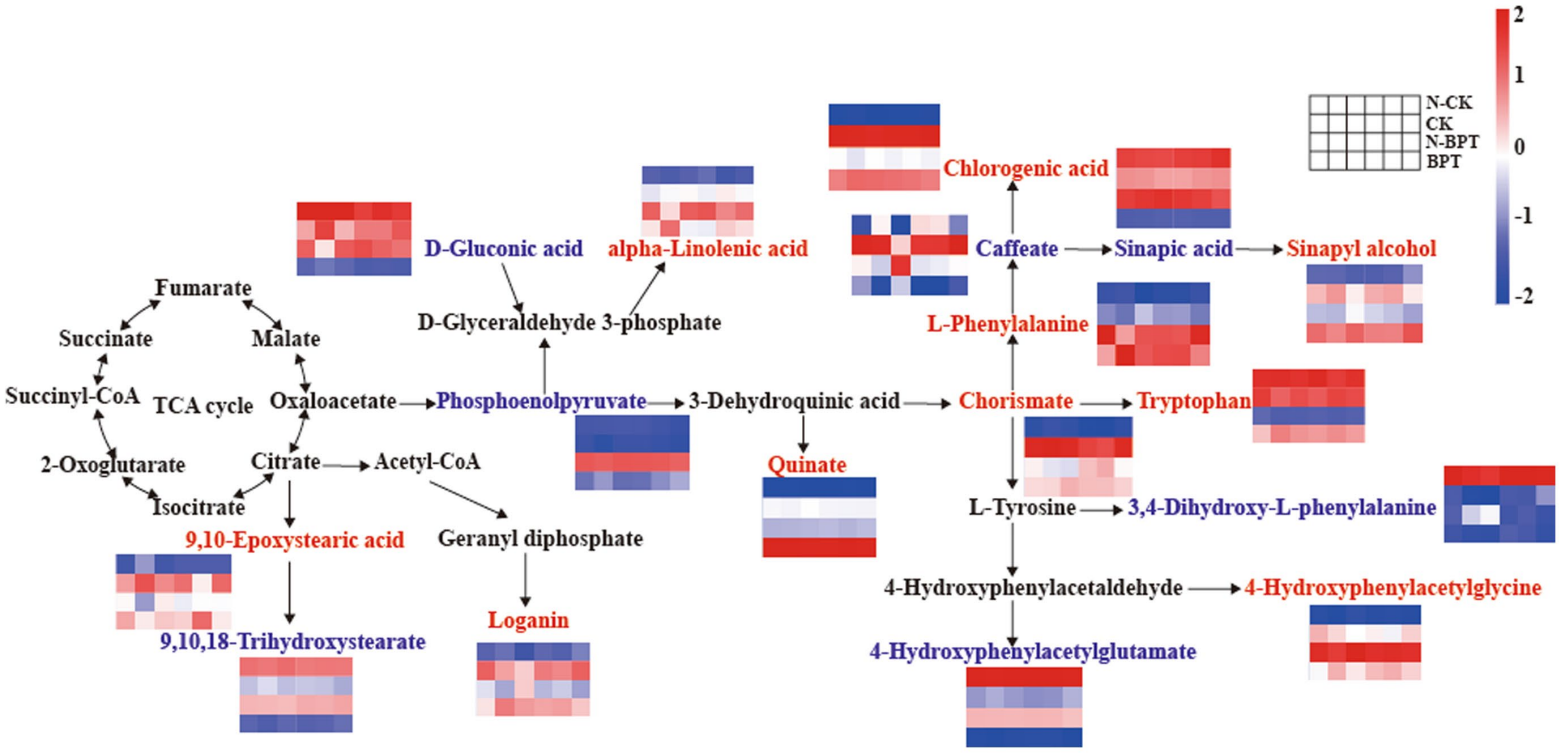
Enrichment pathway analysis of differential metabolite abundance among treatments. Heatmap illustrates the relative abundance of metabolites across different treatments. Metabolites shown in red indicate upregulation, while those in blue indicate downregulation in the BPT treatment.

A comprehensive metabolic pathway map (Figure 8) demonstrated that key altered metabolites were primarily involved in phenylpropanoid biosynthesis, tyrosine metabolism, and phenylalanine, tyrosine, and tryptophan biosynthesis. In the phenylpropanoid biosynthesis pathway, BPT treatment upregulated L-phenylalanine, chlorogenic acid, and sinapyl alcohol, while downregulating caffeate and sinapic acid. Within the phenylalanine, tyrosine, and tryptophan biosynthesis pathway, BPT increased the levels of quinate, chorismate, and tryptophan, but decreased phosphoenolpyruvate. In tyrosine metabolism, both CK and BPT treatments reduced the levels of 3,4-dihydroxy-L-phenylalanine and 4-hydroxyphenylacetylglutamate, whereas the abundance of 4-hydroxyphenylacetylglycine increased. Collectively, these results indicate that biochar, particularly BPT, substantially remodels primary and secondary metabolic pathways in tobacco, promoting the accumulation of phenylpropanoid- and amino acid-related intermediates associated with plant defense and stress adaptation.

## 4 Discussion

### 4.1 Architectural feature of ball-milled phosphorus modified biochar enhance tobacco resistance against *Phytophthora nicotianae*

Biochar is widely recognized as an effective soil amendment make plants more resistant to a variety of insect pests and pathogens (Wang Y. B. et al., 2024; Wang et al., 2025; Yang et al., 2022; Hafeez et al., 2022; Fayyaz et al., 2025). Numerous studies have shown that biochar can effectively mitigate crop diseases such as *Fusarium* wilt, tomato bacterial wilt, and tobacco root rot (Chen et al., 2023; Wang S. et al., 2024; Ge et al., 2023). Fayyaz et al. (2025) found that sugarcane biochar was effective in reducing root-knot nematode infection in tomato crops.

In the present study, ball milling and phosphoric acid (H_3_PO_4_) modification were employed to produce an engineered biochar from tobacco stalk waste. Such physical–chemical modification approaches are known to profoundly alter the microstructure and surface chemistry of biochar, thereby increasing its reactivity and functional diversity (Liu Z. X. et al., 2022). The H_3_PO_4_-modified biochar exhibited higher porosity, a more ordered pore network, and a markedly elevated surface phosphorus content (Figure 1). P plays a central role in plant metabolism, membrane stability, and energy transfer, and its availability can influence the activation of defense responses against biotic stress. Therefore, H_3_PO_4_-modified biochar significantly enhanced nutrient uptake (N, P, K) and growth performance of *Kosteletzkya virginica* (Dai et al., 2025). Similarly, modification of biochar surfaces with various elements or compounds such as Si, SiO_2_ NPs, or thiourea has been shown to enrich functional groups (e.g., Si-O-Si, -OH, or -SOx), increasing the material’s affinity for metals and nutrients while mitigating abiotic and biotic stress in plants (Yuan et al., 2017; Lai et al., 2025; Jin et al., 2023; Peng et al., 2024; Muthusamy et al., 2025). In this study, both H_3_PO_4_ modification and the combined ball milling-H_3_PO_4_ treatment significantly altered the surface chemistry of the original biochar, introducing abundant phosphate (P-O) and hydroxyl (-OH) groups (Figure 2). These newly formed functional groups likely enhanced buffer the plant against pathogen-induced stress. As a result, the ball-milled phosphorus-modified biochar (BPT) markedly promoted tobacco growth and suppressed the expansion of *P. nicotianae* lesions (Figure 4). Taken together, the improved physicochemical characteristics of BPT-namely its higher porosity, enriched P content, and abundant reactive functional groups. These features collectively contribute to enhanced tobacco resistance by mitigating oxidative and pathogen-induced damage.

### 4.2 Ball-milled phosphorus-modified biochar enhances photosynthetic efficiency and antioxidant defense in tobacco

Photosynthesis is a fundamental physiological process that underpins plant growth and productivity, with chlorophyll serving as the primary pigment for light energy capture and conversion. Chlorophyll degradation under environmental or biotic stress severely limits photosynthetic performance and overall plant vitality (Sherin et al., 2022). Previous research has shown that biochar applications can mitigate stress-induced chlorophyll loss and enhance photosynthetic capacity. For instance, Tu et al. (2025) reported a marked increase in chlorophyll concentration in salt-stressed sunflowers following biochar amendment. Consistent with these findings, the present study demonstrated that soil application of ball-milled phosphorus-modified biochar (BPT) significantly increased chlorophyll *a*, chlorophyll *b*, and total chlorophyll contents in tobacco compared with the control (Figures 5A–C), indicating that BPT contributes to improved photosynthetic performance under both normal and pathogen-challenged conditions. Reactive oxygen species (ROS), including hydrogen peroxide (H_2_O_2_) and superoxide anion (O_2_^.-^), are by-products of aerobic metabolism in plants. Under stress conditions, excessive ROS accumulation disrupts cellular homeostasis, leading to lipid peroxidation, membrane damage, and oxidative injury (Liu et al., 2024). Malondialdehyde (MDA), a product of lipid peroxidation, serves as a reliable biomarker of oxidative damage (Bilal et al., 2020). Elevated levels of MDA and H_2_O_2_ have been reported in tomato plants infected with *Fusarium oxysporum*, reflecting oxidative stress (Abdelaziz et al., 2022). In our study, tobacco plants infected with *P. nicotianae* (CK) exhibited the highest concentrations of MDA, H_2_O_2_, and O_2_^.-^, confirming the induction of oxidative stress. However, BPT application markedly reduced these oxidative markers (Figures 5D–F), indicating that the modified biochar effectively mitigated ROS accumulation and lipid peroxidation. This reduction in oxidative stress is consistent with enhanced disease resistance in tobacco. Gao et al. (2023) similarly reported that biochar suppressed ROS production and elevated POD activity in pathogen-infected tomato leaves. Likewise, Wang et al. (2014) found that biochar increased the activities of CAT, SOD, and POD in apple seedlings, alleviating apple replant disease. In our experiment, all biochar treatments significantly enhanced the activities of these antioxidant enzymes (Figures 5G–I), suggesting that biochar enhances tobacco tolerance to *P. nicotianae* by improving photosynthetic pigment stability and strengthening enzymatic ROS-scavenging capacity.

Phytohormones play pivotal roles in coordinating plant growth, development, and defense signaling. IAA promotes cell elongation and root development, while JA serves as a key signal in plant defense against necrotrophic pathogens by activating induced systemic resistance (Mehari et al., 2015; Qin et al., 2023). In this study, both biochar application and pathogen infection significantly altered hormone profiles in tobacco. Compared with the control, biochar-treated plants exhibited elevated IAA and JA contents, with the highest levels observed in inoculated groups (Figures 6A,B). Similar findings were reported by Bisht et al. (2024), who observed increased IAA concentrations in biochar-amended chickpea under drought stress, enhancing root water uptake and stress tolerance. Likewise, Waqas et al. (2018) showed that biochar application promoted JA accumulation in rice, enhancing resistance to the white-backed planthopper. These results suggest that BPT stimulates both growth-promoting and defense-inducing hormonal responses in tobacco under pathogen stress. ABA and SA are central regulators of stress signaling and play critical roles in balancing defense activation and growth processes. Under optimal conditions, plants maintain low basal levels of these hormones, but stress exposure triggers rapid accumulation (Peng et al., 2021). Excessive ABA, however, can induce premature leaf senescence and inhibit photosynthesis (Peng et al., 2021). Previous studies have indicated that plants under low-stress conditions contain relatively high endogenous levels of SA and ABA (Rahimzadeh and Ghassemi-Golezani, 2023; Woo et al., 2025). In this study, tobacco plants treated with BPT exhibited lower ABA accumulation following *P. nicotianae* infection compared with untreated controls (Figure 6D), indicating a reduction in pathogen-induced stress and improved physiological balance. Similarly, SA showed reduced accumulation in inoculated tobacco under BPT and PT treatments (Figure 6C), suggesting alleviation of biotic stress. We speculate that the application of modified biochar enhanced disease resistance in tobacco, thereby reducing the stress level within the plants. JA, SA, and ABA are the primary hormones regulating plant defense responses against pathogens and pests, whereas IAA is primarily considered a growth hormone. SA was involved in regulating the biosynthesis and transport of IAA. High-concentration SA were generally inhibitory to overall root growth, whereas low-level SA can promote the development of the root apical meristem (Koo et al., 2020). In dicotyledonous plants, JA and SA often exhibit an antagonistic relationship in disease defense. The inactivation of the JA receptor results in higher SA levels and enhanced pathogen resistance (Spoel and Dong, 2008). The interaction between IAA and JA, however, can be either synergistic or antagonistic (Mu et al., 2025). Furthermore, SA can enhance freezing tolerance in wheat by inducing endogenous ABA signal (Wang et al., 2018). Plant growth and development are constantly influenced by external conditions. Under biotic or abiotic stress, phytohormones can regulate defense response alone or in combination with other hormones, thereby enabling plants to better mitigate stress. Overall, our results indicate that ball-milled phosphorus-modified biochar enhances tobacco resistance to *P. nicotianae* by maintaining chlorophyll stability, stimulating antioxidant enzyme activity, reducing ROS accumulation, and modulating hormonal balance. These integrated physiological and biochemical adjustments collectively strengthen photosynthetic efficiency and defense capacity, enabling plants to better withstand pathogen-induced stress.

### 4.3 Ball-milled phosphorus-modified biochar enhances phenylpropanoid biosynthesis and metabolic defense against TBS

Plants produce a vast array of metabolites broadly classified as primary and secondary. Primary metabolites such as carbohydrates, lipids, and proteins are essential for growth and energy metabolism through pathways like glycolysis and the TCA cycle (Anzano et al., 2022). In contrast, secondary metabolites, including carotenoids, phenolics, flavonoids, and terpenoids, though not indispensable for survival, play crucial roles in plant defense against biotic and abiotic stresses (Razzaq et al., 2019). In the present study, both biochar application and *P. nicotianae* inoculation significantly altered the metabolic profile of tobacco. The differential metabolites were mainly associated with lipids and lipid-like molecules, phenolic compounds, organic acids, and amino acids, indicating coordinated metabolic reprogramming in response to stress.

Within the phenylpropanoid biosynthesis and phenylalanine, tyrosine, and tryptophan biosynthesis pathways, BPT treatment notably upregulated tryptophan and L-phenylalanine levels (Figure 8). Tryptophan stimulates auxin biosynthesis in the rhizosphere, thereby promoting plant growth and stress tolerance (Mayo-Prieto et al., 2019). Similarly, Miao et al. (2019) demonstrated that activation of the tryptophan synthesis pathway enhanced Verticillium dahliae resistance in cotton. Phenylalanine serves as a precursor for monolignol synthesis via the phenylpropanoid pathway, and monolignols are the building blocks of lignin (Lee et al., 2019). Lignin deposition strengthens cell walls, forming a physical barrier against pathogens and serving as an integral component of plant immunity (Cesarino, 2019; Adobor et al., 2023). Compared with the N-CK group, BPT treatment significantly increased the abundance of phenolic acids, particularly chlorogenic acid (Figure 8). Phenolic acids play dual roles in plant-microbe interactions: mediating signaling and providing antioxidant protection under stress conditions (Kaur and Suseela, 2020). Chlorogenic acid has been identified as a key resistance factor, enhancing tolerance to multiple biotic and abiotic stresses (Atanasova-Penichon et al., 2012; Sabino et al., 2019). For example, Si treatment increased chlorogenic acid accumulation in rose leaves, correlating with reduced mildew powdery incidence (Shetty et al., 2011), while higher chlorogenic acid levels were also observed in resistant wheat cultivars infected by *Fusarium graminearum* (Liu C. X. et al., 2022). The upregulation of chlorogenic acid in BPT-treated plants suggests that ball-milled H_3_PO_4_-modified biochar effectively activates defense-related secondary metabolism, enhancing resistance against *P. nicotianae*. In response to pathogen-induced oxidative stress, plants activate defense systems comprising enzymatic and non-enzymatic antioxidants (Colak et al., 2021). Key enzymatic components include CAT, POD, and SOD, while phenolic compounds (e.g., phenolic acids) represent non-enzymatic constituents. Antioxidant compounds maintain the dynamic balance between the production and scavenging of reactive oxygen species (ROS) under stress conditions (Gudkov et al., 2019). Notably, during plant-pathogen interaction, increase in peroxidase activity is often associated with the accumulation of phenolic compounds in the cell wall, collectively enhancing the plant’s disease resistance (Jaiti et al., 2007). Additionally, phosphoenolpyruvate (PEP) serves as a precursor to pyruvate (Dumont and Rivoal, 2019). Fan et al. (2023) reported that PEP is catalyzed by phosphoenolpyruvate carboxylase (PEPC) to form oxaloacetate, an intermediate of the TCA cycle, thereby supporting energy metabolism under stress. Enhanced TCA cycle activity provides additional energy for defense responses and growth under pathogen attack (Xiao et al., 2022). In the present study, organic acids such as chorismate and 4-hydroxyphenylacetylglycine also accumulated under BPT treatment. Chorismate is a central intermediate leading to both plant hormone synthesis and aromatic amino acid formation (Yuan et al., 2022). The accumulation of organic acids is often associated with improved pathogen resistance (Zhao et al., 2023), and similar patterns have been observed in wild soybean exhibiting tolerance to alkaline stress (Sun et al., 2025). Pathogen infection typically compromises cell membrane integrity (Zhang et al., 2024). Lipids play a dual role in maintaining membrane stability and scavenging reactive oxygen species (ROS), thus functioning as antioxidant protectants (Zhu et al., 2024). The disease resistance of plants may be influenced by the antioxidant property of lipids and play a significant role in plant-pathogen interaction. In this study, BPT treatment significantly upregulated several lipid-related metabolites, including alpha-linolenic acid, loganin, and 9,10-epoxystearic acid. Alpha-linolenic acid not only a polyunsaturated fatty acid but also as a precursor of JA, a key defense hormone involved in plant stress responses (Zi et al., 2022). JA is found in the photoreceptors of plants, specifically in the membrane lipids of chloroplast. Alpha-Linolenic acid serves to activate the expression of JA-related defense genes and contributes to maintaining cell membrane integrity during pathogen attack (Wasternack and Song, 2017). Furthermore, a higher level of unsaturated fatty acids can increase the flexibility of cell membranes, thereby enhancing the capacity of cells to withstand pathogen infection (Zhang et al., 2024). Collectively, these findings suggest that ball-milled phosphorus-modified biochar (BPT) enhances tobacco resistance to *P. nicotianae* by promoting phenylpropanoid biosynthesis, increasing the accumulation of phenolic acids, amino acids, and lipids, and enhancing energy metabolism. This comprehensive metabolic reprogramming strengthens antioxidant capacity and defense signaling, thereby improving overall plant health and resilience against pathogen invasion.

Modified biochar demonstrates a positive role in enhancing tobacco disease resistance. As an integrated system, the interactions between biochar, soil, and tobacco are complex and interconnected. While this study primarily focused on the effects of biochar on the physiological and biochemical characteristics of tobacco, the role of soil-particularly its microbial community-warrants further investigation. The application of biochar to soil contributes to soil health by enhancing the abundance of beneficial microorganisms, suppressing the reproduction of soil-borne pathogens and pests, and strengthening plant resistance against diseases and pests (Martínez-Gómez et al., 2023; Waqar et al., 2025; Yan et al., 2024; Li et al., 2022). Therefore, future research should prioritize examining how modified biochar influences the structure of soil microbial communities in the context of soil-borne disease management. Additionally, as this experiment was a pot-based simulation, subsequent research necessitates field-scale validation. The selection of appropriate modified biochar formulations for disease management must be informed by specific soil, climatic, and agronomic conditions to achieve optimal disease control.

## 5 Conclusion

Ball milling combined with phosphorus modification significantly enhanced the structural and chemical properties of biochar and markedly increased surface phosphorus content. The modified biochar (BPT) surface was enriched with functional groups including -OH, P-OH, and P-O, which improved its reactivity and soil interaction potential. BPT application in soil effectively enhanced tobacco chlorophyll accumulation, strengthened plant antioxidant defense systems, and modulated phytohormone balance. Moreover, BPT upregulated the biosynthesis of amino acids and phenolic acids through the phenylpropanoid and phenylalanine-tyrosine-tryptophan pathways, as well as influencing lipid metabolism to improve resistance of *Nicotiana tabacum* to *P. nicotianae* infection.

## Data Availability

The original contributions presented in the study are included in the article/Supplementary material, further inquiries can be directed to the corresponding authors.
