# Supplementary material for: Ball milled phosphorus modified biochar improved Nicotiana tabacum L. resistance against Phytophthora nicotianae: reducing oxidative damage, increasing defense hormone content and promoting phenylpropanoid metabolism
Source: Front Microbiol. 2026 Jan 12;16:1734991. doi: 10.3389/fmicb.2025.1734991 (PMC12832675; doi:10.3389/fmicb.2025.1734991)
Supplement: Supplementary file 1 [file Supplementary_file_1.docx]

**Supporting Information**

**Ball milled phosphorus modified biochar improved *Nicotiana tabacum* L. resistance against *Phytophthora nicotianae*：reducing oxidative damage, increasing defense hormone content and promoting phenylpropanoid metabolism**

**Chunlan Ming^1^, Yushuai Zhang^1^,** **Mengze Li^1^, Mohamed G Moussa^3^, Tengfei Liu^1^,** **Hang Wang^1^,** **Yongfei Ma^2^,** **Wuxing Huang^1^,** **Zicheng Xu^1^,** **Jiayang Xu^2*^,** **Wei Jia^1*^**

^1^ Key Laboratory for Tobacco Cultivation of Tobacco Industry, National Tobacco Cultivation and Physiology and Biochemistry Research Center, College of Tobacco Science, Henan Agricultural University, Zhengzhou 450046, China

^2^ College of Resources and Environment, Henan Agricultural University, Zhengzhou 450046, China

^3^ Soil and Water Research Department, Nuclear Research Center, Egyptian Atomic Energy Authority, Cairo 13759, Egypt

Corresponding author and E-mail:

Wei Jia: [jiawei@henau.edu.cn](mailto:jiawei@henau.edu.cn); Jiayang Xu: jiayangxu@henau.edu.cn

**Text. S1. Preparation of Biochar**

**Text. S2. Basic physicochemical properties of the experimental soil**

**Text. S3. Physico-chemical characterization analysis of biochar**

**Text. S4. The detailed parameters of the test kits**

**Text. S5. LC-MS experimental instruments and parameters**

**Table. S1. The specific surface area and average pore size of the biochars**

**Table. S2. Formula, m/z, RT,and CAS of the metabolites in Fig. 8**

**Fig. S1. N_2_ adsorption-desorption isotherms and pore size distribution of T, BT, PT and BPT**

**Fig. S2. Phenotypic profiles of tobacco under different biochar treatments.**

**Fig. S3. Effects of different biochar treatments on the TBS lesion length**

**Fig. S4. Pie chart of the classes of metabolites identified**

**Fig. S5. Partial least squares discriminant analysis of different treatment groups**

**Fig. S6. Quantification of metabolites**

**Text. S1. Preparation of Biochar.**

An appropriate amount of T was placed in a hydrothermal reaction kettle containing 1 mol/L MgCl_2_ solution and 1 mol/L CaCl_2_ solution, respectively. stirred in a constant-temperature water bath at 25°C for 12 hours, and then dried to a constant weight in a blast dryer at 80°C. Then, it underwent secondary pyrolysis at 600°C for 2 hours (with a heating rate of 10°C/min and a N₂ flow rate of 0.2 L/min). After cooling, cleaning with ultrapure water, drying, grinding, and sieving, MgCl_2_-modified biochar (MgT) and CaCl_2_-modified biochar (CaT) were obtained.

Finally, An appropriate amount of MgT and CaT were placed in a planetary ball mill (Model: XQM-0.4 A, Brand: Tencan Power (TianChuang Powder), Changsha, China) and ball-milled at 500 rpm for 120 minutes. The mass ratio of stainless steel balls to TBC was 20:1. The resulting biochar was labeled as MgCl_2_-modified tobacco stalk biochar (BMgT) and CaCl_2_-modified tobacco stalk biochar (BCaT).

**Text. S2. Basic physicochemical properties of the experimental soil.**

The experimental soil had the following basic physicochemical properties: pH 8.51, organic matter 12.4 g/kg, alkaline nitrogen 37.94 mg/kg, available phosphorus 5.31 mg/kg, and available potassium 96 mg/kg.

**Text. S3. Physico-chemical characterization analysis of biochar.**

The surface morphology of T, BT, PT, and BPT were characterized using scanning electron microscopy (SEM-EDS, ZEISS, Sigma 300, UK). Functional groups were identified by Fourier transform infrared spectroscopy (FTIR, IRTracer 100, Japan), surface elemental composition was analyzed by X-ray photoelectron spectroscopy (XPS, Thermo Fisher Scientific-K-Alpha, USA) and Brunauer-Emmett-Teller (BET, Micromeritics-ASAP 2420, USA) was used for the detection of specific surface area and pore structure of biochars.

**Table. S1. The specific surface area and average pore size of the biochars**

| Thermophysical properties | T | BT | PT | BPT |
| --- | --- | --- | --- | --- |
| Surface Area(m^2^/g) | 1.6860±0.3575b | 9.4022±0.1631a | 0.4311±0.0574c | 0.7111±0.0054c |
| Average pore size (nm) | 21.6098±0.2599b | 12.5232±0.9957c | 30.2524±1.6440a | 20.2093±2.0571c |

**Fig. S1. N_2_ adsorption-desorption isotherms and pore size distribution of T, BT, PT and BPT.**


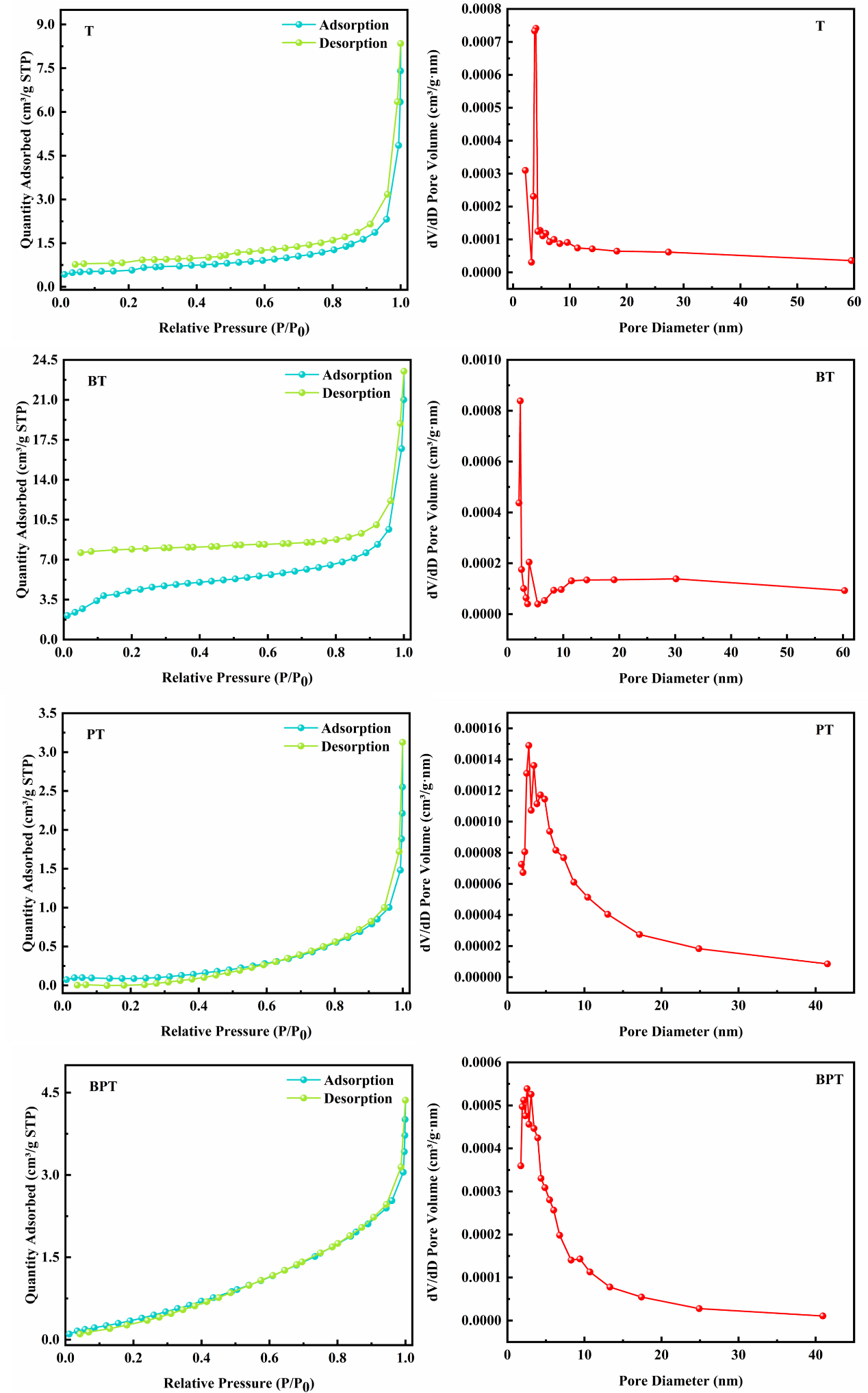


**Fig. S2. Phenotypic profiles of tobacco under different biochar treatments.**


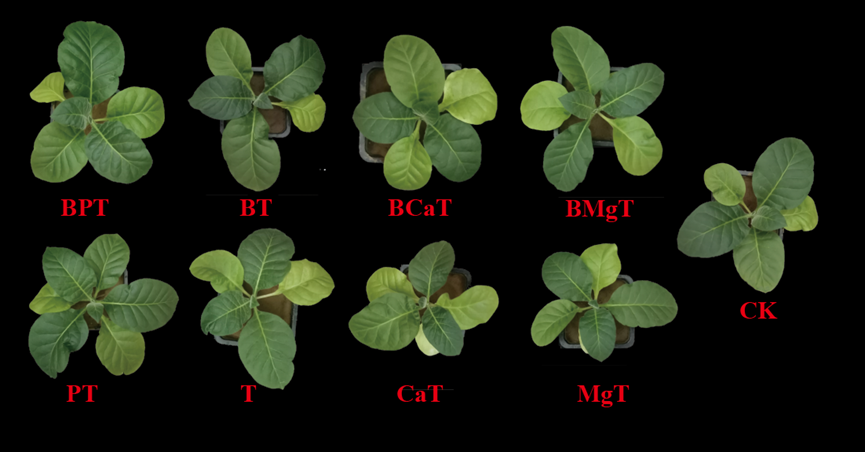


**Fig. S3. Effects of different biochar treatments on the TBS lesion length.**
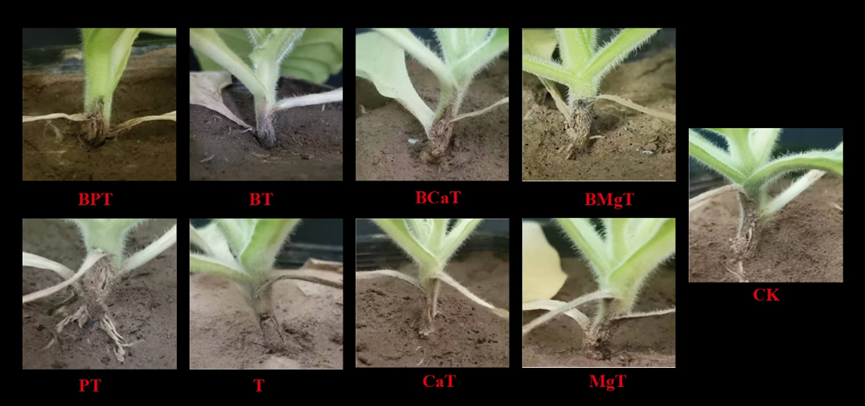


**Text. S4. The detailed parameters of the test kits**

**1. Enzymatic activity and reactive oxygen species indices**

| Parameter | CAT | POD | SOD | MDA | H_2_O_2_ | O_2_^.-^ |
| --- | --- | --- | --- | --- | --- | --- |
| Catalog number | CAT-1-W | POD-1-Y | SOD-1-Y | MDA-1-Y | H2O2-1-Y | SA-1-G |

**2.** **Hormone-related indicators**

(1)IAA

Kit catalog: YJ147100

Minimum detection concentration:＜1.0nmol/L

Standard curve:

| Concentration(nmol/L) | 0 | 3 | 6 | 12 | 24 | 48 |
| --- | --- | --- | --- | --- | --- | --- |
| Optical Density | 0.0495 | 0.7657 | 1.3711 | 2.1209 | 2.904 | 3.2774 |


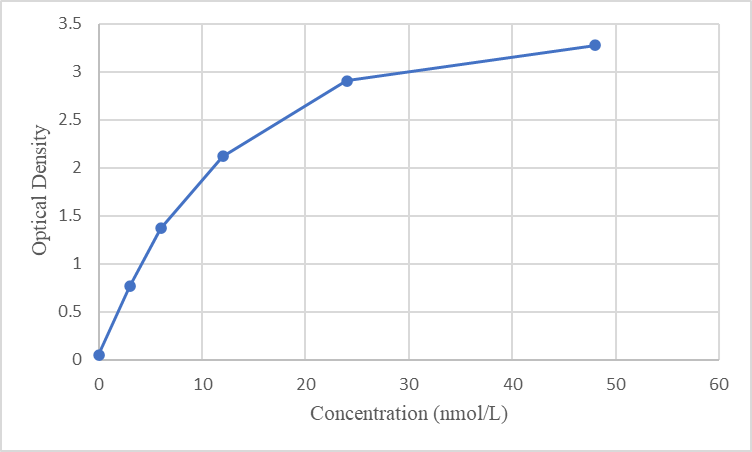


(2)JA

Kit catalog: YJ690034

Minimum Detection Concentration: ＜10pg/mL

Standard curve:

| Concentration(pg/mL) | 0 | 250 | 500 | 1000 | 2000 | 4000 |
| --- | --- | --- | --- | --- | --- | --- |
| Optical Density | 0.0655 | 0.2071 | 0.3183 | 0.4972 | 1.0835 | 1.2508 |


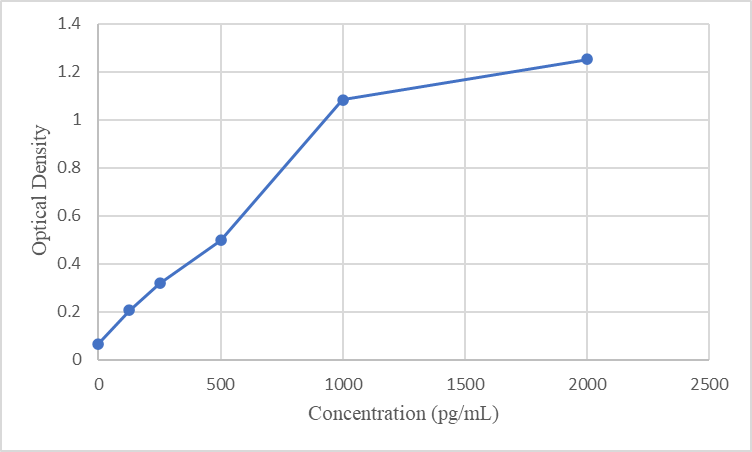


(3)SA

Kit catalog: YJ291089

Minimum Detection Concentration: ＜1.0ng/mL

Standard curve:

| Concentration(ng/mL) | 0 | 1.5 | 3 | 6 | 12 | 24 |
| --- | --- | --- | --- | --- | --- | --- |
| Optical Density | 0.0599 | 0.5176 | 0.7982 | 1.3809 | 2.4266 | 3.2086 |


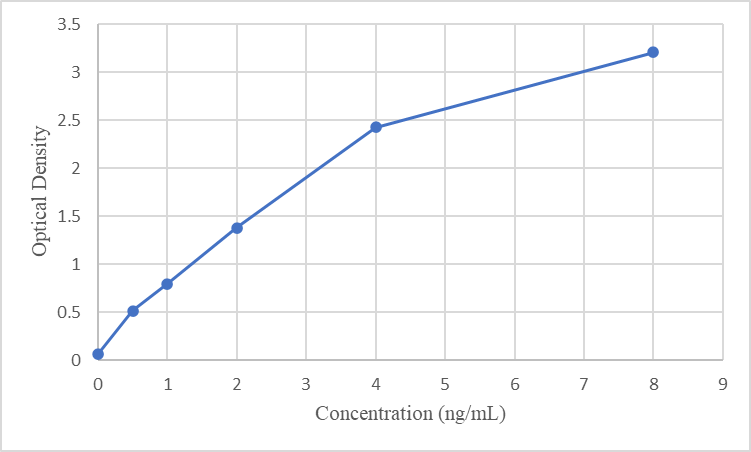


(4)ABA

Kit catalog: YJ034754

Minimum Detection Concentration: ＜1.0ng/mL

Standard curve:

| Concentration(ng/mL) | 0 | 5 | 10 | 20 | 40 | 80 |
| --- | --- | --- | --- | --- | --- | --- |
| Optical Density | 0.0425 | 0.5282 | 0.827 | 1.547 | 2.5449 | 3.0918 |


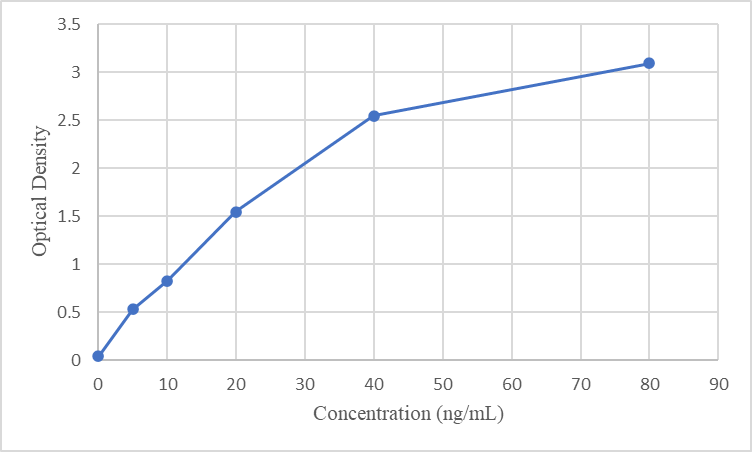


**Text. S5. LC-MS** **experimental instruments and parameters**

(1) Instrumental parameters

| Instrument types | Model | Brand | Country of origin |
| --- | --- | --- | --- |
| Chromatograph | VanquishUHPLC | Thermo Fisher | Germany |
| Mass Spectrometer | QExactive™HF | Thermo Fisher | Germany |
| Chromatographic column | Hypesil Gold column  (100×2.1mm,1.9μm) | Thermo Fisher | USA |

(2) Chromatography Parameters

Chromatographic column: Hypersil Gold column(C18)

Column temperature: 40℃

Flow rate: 0.2mL/min

Mobile phaseA: 0.1% formic acid

Mobile phase B: methanol

(3) Chromatographic elution program

| Time | A% | B% |
| --- | --- | --- |
| 0 | 98 | 2 |
| 1.5 | 98 | 2 |
| 3 | 15 | 85 |
| 10 | 0 | 100 |
| 10.1 | 98 | 2 |
| 11 | 98 | 2 |
| 12 | 98 | 2 |

(4) Mass spectrometry parameters

Scans mass range: m/z100-1500; ESI source parameters were set as follows: Spray Voltage: 3.5kV; Sheath gas flow rate: 35psi; Aux Gas flow rate: 10L/min; Capillary Temp: 320°C; S-lens RF level: 60; Aux gas heater temp: 350°C; Polarity: positive, negative; MS/MS secondary scan was performed in data-dependent scans.

**Fig. S4. Pie chart of the classes of metabolites identified**
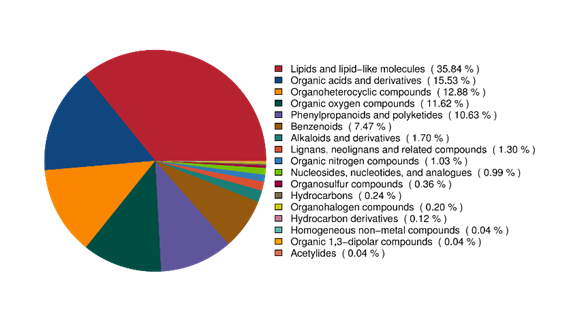


**Fig. S5. Partial least squares discriminant analysis of different treatment groups**

**
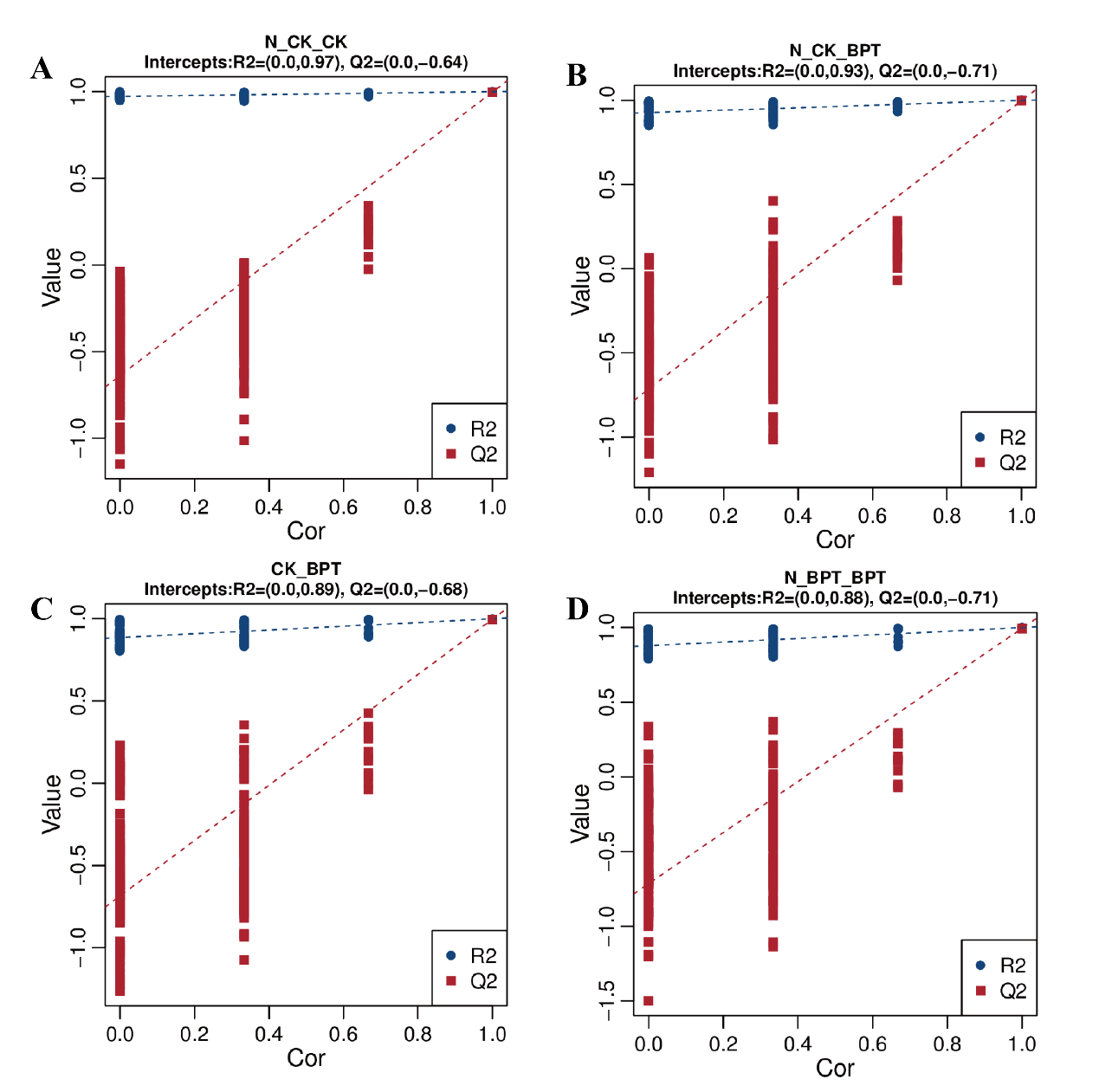
**

**Table. S2.** **Formula, m/z, RT,and CAS of the metabolites in Fig. 8**

| Name | Formula | m/z | RT (min) | CAS |
| --- | --- | --- | --- | --- |
| Chlorogenic acid | C16H18O9 | 377.0847964 | 5.268 | 327-97-9 |
| L-Phenylalanine | C9H11NO2 | 166.086361 | 5.013 | 63-91-2 |
| Chorismate | C10H10O6 | 227.0551135 | 4.716 | 617-12-9 |
| Tryptophan | C11H12N2O2 | 203.0820979 | 5.293 | 73-22-3 |
| Caffeate | C9H8O4 | 163.0395124 | 5.282 | 501-16-6 |
| Sinapinic acid | C11H12O5 | 223.0608775 | 5.312 | 530-59-6 |
| Sinapyl alcohol | C11H14O4 | 211.0968526 | 5.068 | 537-33-7 |
| alpha-Linolenic acid | C18H30O2 | 279.2317009 | 7.974 | 463-40-1 |
| Loganin | C17H26O10 | 413.1420531 | 5.241 | 18524-94-2 |
| Quinate | C7H12O6 | 191.0553136 | 1.463 | 77-95-2 |
| 9,10-Epoxystearic acid | C18H34O3 | 299.2584423 | 9.156 | 2443-39-2 |
| 9,10,18-Trihydroxystearate | C18H36O5 | 331.2491264 | 6.883 |  |
| Phosphoenolpyruvate | C3H5O6P | 226.9959768 | 1.49 | 138-08-9 |
| D-Gluconic Acid | C6H12O7 | 219.0477588 | 6.135 | 526-95-4 |
| 3,4-Dihydroxy-L-phenylalanine | C9H11NO4 | 198.0766056 | 5.47 | 59-92-7 |
| 4-Hydroxyphenylacetylglutamate | C13H15NO6 | 280.0827431 | 5.763 |  |
| 4-Hydroxyphenylacetylglycine | C10H11NO4 | 232.0584856 | 5.131 | 28116-23-6 |

**Fig. S6. Quantification of Chlorogenic acid, L-phenylalanine, Tryptophan, Alpha-linolenic acid
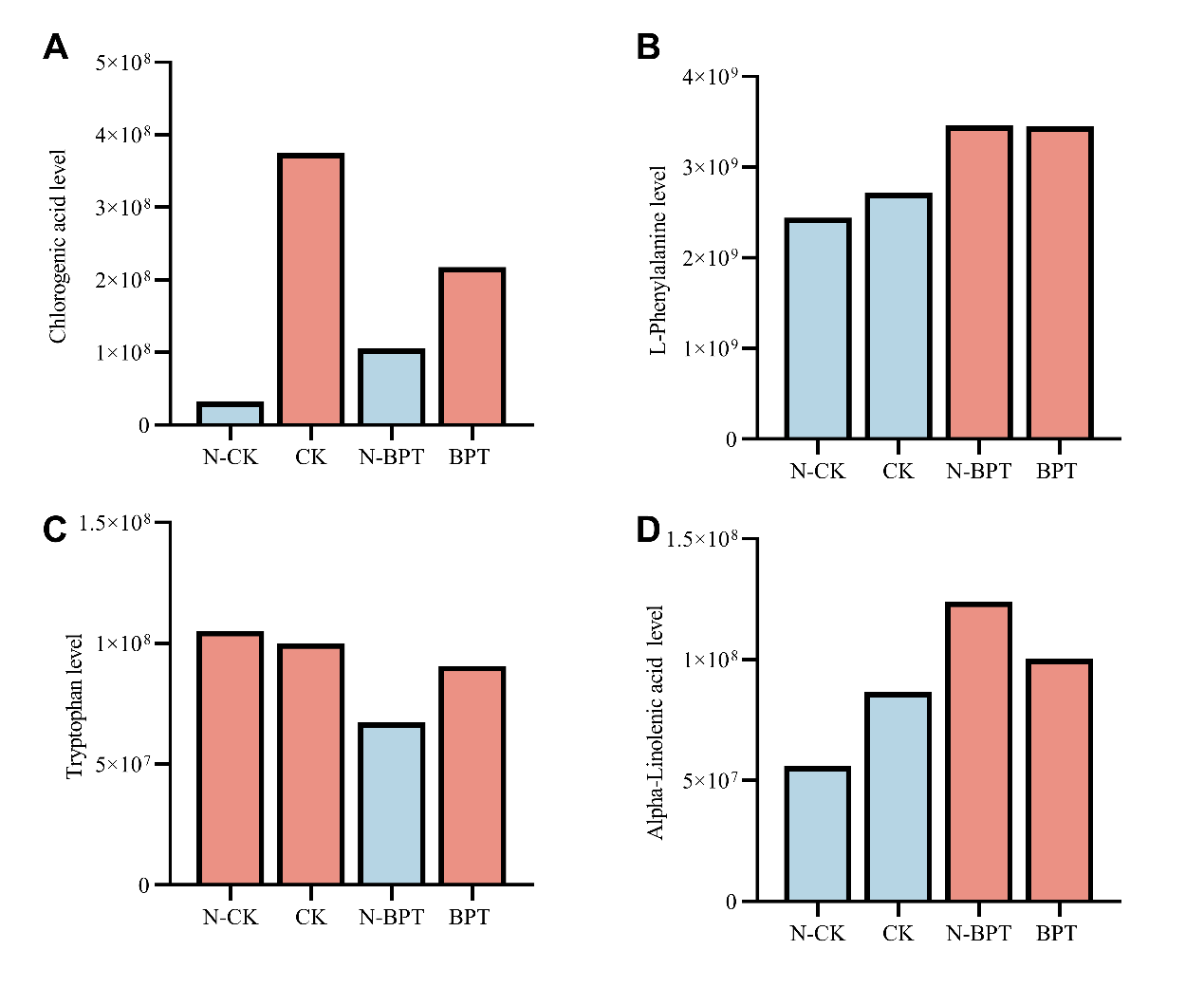
**
